# Transient neuropsychiatric events after HPV vaccination among adolescent schoolgirls in India

**DOI:** 10.6026/973206300220518

**Published:** 2026-01-31

**Authors:** Raj Kamal Choudhary, Obaid Ali, Rakhshanda Khatoon

**Affiliations:** 1 Department of Medicine, Jawaharlal Nehru Medical College, Bhagalpur, India

**Keywords:** HPV vaccine, AEFI, anxiety reaction, absence seizure, vaccine safety

## Abstract

Clusters of transient neuropsychiatric symptoms following HPV vaccination among adolescents, particularly in school-based immunisation settings, can lead to diagnostic uncertainty, unnecessary medical interventions and public concern regarding vaccine safety. We describe a cluster of five 13-year-old schoolgirls who developed acute dizziness, brief loss of consciousness, hyperventilation and anxiety shortly after vaccination, despite normal examinations and unremarkable investigations. The clinical pattern was most consistent with vasovagal and anxiety-related reactions, although absence seizures could not be fully excluded without EEG. All recovered rapidly with reassurance, hydration and short-term symptomatic management. Awareness of such benign, self-limiting events can prevent unnecessary interventions and support vaccine confidence.

## Background:

The human papillomavirus (HPV) vaccine is recommended globally for the prevention of cervical cancer and other HPV-related diseases [1, 2]. It is generally safe and well tolerated, with extensive post-licensure surveillance and cohort event-monitoring studies demonstrating a favourable safety profile across different settings [1, 3 and 4]. Large real-world studies have shown that most adverse events following immunisation (AEFI) are non-serious and self-limiting, commonly including dizziness, syncope and anxiety-related reactions, without evidence of new or unexpected safety signals [1, 5 and 6]. Clinical trials and observational studies of bivalent, quadrivalent and 9-valent HPV vaccines further confirm good tolerability and sustained immunogenicity in adolescents and young adults [7, 8]. However, transient neuropsychiatric and vasovagal symptoms may occasionally occur, particularly when vaccinations are administered in school-based or mass-immunisation settings and can lead to emergency visits and public concern when cases occur in clusters [9, 10]. Therefore, it is of interest to report on the transient neuropsychiatric events after HPV vaccination among adolescent schoolgirls in India.

## Methods:

We report a series of five adolescent schoolgirls who developed neuropsychiatric symptoms shortly after receiving CERVAVAC, a quadrivalent HPV vaccine containing HPV types 6, 11, 16 and 18. This observational study retrospectively reviewed five consecutive 13-year-old students who were vaccinated in a school setting in Banka district, Bihar and subsequently presented to JLNMCH, Bhagalpur. Data on demographics, clinical features, investigations, management and outcomes were collected and analysed.

## Observations:

We observed five 13-year-old adolescent schoolgirls who developed acute-onset symptoms following administration of the HPV vaccine during a school-based immunisation session. The most common presenting complaints included dizziness, nausea, headache, hyperventilation and multiple episodes of transient loss of consciousness occurring either immediately or within two days of vaccination. None of the patients exhibited seizure activity, focal neurological deficits, or abnormalities on MRI brain imaging. Vital signs on presentation were stable in all cases and routine laboratory investigations were within normal limits. Initial management at local healthcare facilities commonly involved supplemental oxygen, intravenous fluids, ondansetron and antihistamines, after which all patients were referred to our tertiary centre. In-hospital care consisted of supportive management with intravenous fluids, antiemetics, antihistamines, occasional corticosteroids, betahistine and short-term use of clonazepam, propranolol, or escitalopram following psychiatric evaluation. ENT assessment, including the Dix-Hallpike manoeuvre, was negative for vestibular pathology in all patients. All patients experienced complete symptom resolution within 3-4 days of admission, with no residual deficits at discharge. A two-week follow-up confirmed sustained recovery without recurrence.

## Results and Discussion:

The demographic and vaccination-related details of all five cases are summarized in Table 1. All patients were 13-year-old female students who received CERVAVAC in a school-based vaccination session. Table 1 outlines each case with respect to past medical history, vaccine dose number, manufacturer and batch information, vaccine vial details, session site and whether the events occurred as part of a cluster. Timing of symptom onset, hospitalization details and duration of hospital stay are also presented. The investigation findings are provided in Table 2. Routine laboratory tests (CBC, LFT, KFT), ECG, viral serology (HBsAg and anti-HCV), abdominal ultrasound and MRI brain were largely within normal limits for all five patients. These five cases illustrate a consistent pattern of transient neuropsychiatric symptoms-primarily fainting, dizziness, hyperventilation and anxiety-in adolescent girls following HPV vaccination. All patients were managed symptomatically, with psychiatric support and reassurance playing a significant role in recovery. The clinical features and rapid resolution strongly suggest vasovagal syncope and anxiety-related reactions as the most plausible mechanisms. Although the possibility of unrecognized absence seizures cannot be entirely excluded, none of the patients exhibited convulsive activity, post-ictal confusion, or other seizure markers. MRI and laboratory evaluations were unremarkable; however, the absence of EEG assessment limits a definitive exclusion of seizure phenomena. Cluster events in school-based vaccination settings can also precipitate psychogenic responses and hyperventilation. Extensive global research and post-marketing surveillance consistently demonstrate that HPV vaccines (bivalent, quadrivalent and nonavalent) are safe and well tolerated. Most adverse events are mild and short-lived, including injection-site pain, swelling, headache, fatigue, dizziness, nausea, or low-grade fever. Syncope is known to occur in adolescents due to vasovagal reactions, which is why a 15-minute observation period post-vaccination is recommended. Serious reactions such as anaphylaxis are extremely rare (approximately 1-3 cases per million doses). Large pharmacovigilance systems have found no increased risk of Guillain-Barré syndrome, venous thromboembolism, seizures, Complex Regional Pain Syndrome, Postural Orthostatic Tachycardia Syndrome (POTS), or premature ovarian insufficiency. Reports suggesting associations with conditions such as POTS or chronic fatigue syndrome remain unsubstantiated, with no proven causal links. Major global health authorities, including the WHO, reaffirm that HPV vaccines are "extremely safe." These cases emphasise the importance of prompt recognition and supportive care, which help prevent unnecessary interventions and reduce anxiety among patients and caregivers. All patients recovered fully within 3-4 days, reaffirming the benign and self-limiting nature of these reactions. Management commonly included anxiolytics (*e.g.*, clonazepam), antihistamines (*e.g.*, pheniramine), betahistine, IV fluids and in select cases, short courses of antidepressants (escitalopram) or beta-blockers.

## Abbreviations:

CBC-complete blood count;

LFT-liver function test;

KFT-kidney function test;

ECG-electrocardiogram;

HbsAg-Hepatitis B surface antigen;

Anti HCV-Antibody to Hepatitis C virus;

USG-Ultrasonography;

MRI-Magnetic resonance imaging;

WNL-within normal limits;

N/A-not available (propranolol).

Multidisciplinary evaluation, including psychiatric and ENT assessments, provided reassurance and helped exclude organic pathology. Continued follow-up is advisable to monitor for any delayed or persistent symptoms and potential long-term sequelae.

## Conclusion:

We show that transient neuropsychiatric symptoms such as dizziness, fainting, hyperventilation, nausea and anxiety may occur in adolescent girls following school-based HPV vaccination but are benign and self-limiting. All episodes resolved with supportive care and reassurance, with no evidence of underlying organic pathology. The clustered occurrence of these events suggests anxiety-related and vasovagal mechanisms, underscoring the importance of anticipatory guidance and structured post-vaccination observation in school immunization programmes. Implementation of simple preventive measures can minimize anxiety-related reactions, prevent syncope-related injuries and help maintain public confidence in HPV vaccination.

## Advancement to knowledge:

Transient neuropsychitric symptoms following HPV vaccination may occur as anxiety-related adverse events following immunisation (AEFI), particularly in clustered, school-based settings. This report provides Indian programmatic evidence that such events are benign and self-limiting, resolving with reassurance and minimal intervention. Recognising these patterns can help clinicians and public health teams avoid unnecessary investigations and hospitalisation, while supporting vaccine confidence and safe implementation of HCV immunisation programmes.

## Author contributions:

Dr. Raj Kamal Choudhary conceptualized the study, collected clinical data and performed literature review. Dr. Obaid Ali and Dr. Rakhshanda Khatoon assist in data analysis and drafting the manuscript. All the authors contributed to manuscript revision, provided critical input for discussion and approved the final version of the manuscript.

## Ethical approval:

Informed consent was obtained from the patients' legal guardians for publication of their clinical details. All efforts were made to ensure patient anonymity.

No pre-print of this manuscript has been published or posted elsewhere.

## Ethical approval:

Informed consent was obtained from the patients' legal guardians for publication of their clinical details. All efforts were made to ensure patient anonymity.

## Figures and Tables

**Table 1:** Data Collected- Demographic, clinical and vaccination-related characteristics of adolescent girls with transient neuropsychiatric events following HPV vaccination

	**Case 1**	**Case 2**	**Case 3**	**Case 4**	**Case 5**
Age/Sex	13 year/Female	13 year/Female	13 year/Female	13 year/Female	13 year/Female
Past medical history	N/A	N/A	N/A	N/A	N/A
Vaccine	HPV Vaccine: Cervavac	HPV Vaccine: Cervavac	HPV Vaccine: Cervavac	HPV Vaccine: Cervavac	HPV Vaccine: Cervavac
Dose no. given	1st	1st	1st	1st	1st
Name of Manufacturer	Serum Institute of India Pvt. Ltd.	Serum Institute of India Pvt. Ltd.	Serum Institute of India Pvt. Ltd.	Serum Institute of India Pvt. Ltd.	Serum Institute of India Pvt. Ltd.
Batch/Lot no.	2204Z022	2204Z022	2204Z022	N/A	N/A
Mfg. date	Sep-24	Sep-24	Sep-24	N/A	N/A
Exp. date	Oct-27	Oct-27	Oct-27	N/A	N/A
Vaccine vial opening	2/8/2025	2/8/2025	2/8/2025	2/8/2025	2/8/2025
Session site	A.M.S. Amarpur Boys	A.M.S. Amarpur Boys	A.M.S. Amarpur Boys	A.M.S. Amarpur Boys	A.M.S. Amarpur Boys
Source of vaccine	Government supply	Government supply	Government supply	Government supply	Government supply
Case Interviewed	02.08.2025	02.08.2025	02.08.2025	04.08.2025	04.08.2025
Type of Adverse Event (Serious/Severe)	Serious	Serious	Serious	Serious	Serious
Is this part of a cluster?	Yes	Yes	Yes	Yes	Yes
Date & Time of Primary Hospitalization in Referral Hospital, Amarpur, Banka	02.08.2025 2pm	02.08.2025 02:08pm	02.08.2025 02:10pm	04.08.2025 3pm	04.08.2025 3:10pm
Date & Time of Admission in JLNMCH,Bhagalpur	02.08.2025 10:30pm	02.08.2025 11:10pm	02.08.2025 11:11pm	04.08.2025 6:50pm	04.08.2025 7pm
Number of days of hospital stay	3	3	3	3	3
Abbreviations: N/A-not available;
HPV-Human Papilloma Virus;
Mfg. -manufacturing;
Exp. -expiry;
JLNMCH-Jawaharlal Nehru Medical
College and Hospital; Serious
adverse event classification as
per national AEFI surveillance guidelines.

**Table 2:** Laboratory, imaging and diagnostic investigation findings in cases with transient neuropsychiatric symptoms following HPV vaccination

	**Case 1**	**Case 2**	**Case 3**	**Case 4**	**Case 5**
CBC	WNL	WNL	WNL	WNL	WNL
LFT	WNL	WNL	WNL	WNL	WNL
KFT	WNL	WNL	WNL	WNL	WNL
ECG	Normal Study	Normal Study	Normal Study	Normal Study	Normal Study
HBsAg	Non Reactive	Non Reactive	Non Reactive	Non Reactive	Non Reactive
Anti HCV	Non Reactive	Non Reactive	Non Reactive	Non Reactive	Non Reactive
USG Whole Abdomen	Normal Study	Normal Study	Normal Study	Normal Study	Normal Study
MRI Brain	Normal Study	Normal Study	Normal Study	N/A	N/A
